# Implementing Whole-Genome Sequencing for Ongoing Surveillance of Antimicrobial Resistance: Exemplifying Insights Into *Klebsiella pneumoniae*

**DOI:** 10.1093/cid/ciab795

**Published:** 2021-11-27

**Authors:** David M Aanensen, Celia C Carlos, Pilar Donado-Godoy, Iruka N Okeke, K L Ravikumar, NIHR Global Health Research Unit on Genomic Surveillance of Antimicrobial Resistance, Khalil Abudahab, Monica Abrudan, Silvia Argimón, Harry Harste, Mihir Kekre, Dawn Muddyman, Ben Taylor, Anthony Underwood, Nicole Wheeler, Sophia David, Johan Fabian Bernal, Alejandra Arevalo, Maria Fernanda Valencia, Erik C D Osma Castro, Geetha Nagaraj, Varun Shamanna, Vandana Govindan, Akshata Prabhu, D Sravani, M R Shincy, Steffimole Rose, K N Ravishankar, Anderson O Oaikhena, Ayorinde O Afolayan, Jolaade J Ajiboye, Erkison Ewomazino Odih, Marietta L Lagrada, Polle Krystle V Macaranas, Agnettah M Olorosa, June M Gayeta, Elmer M Herrera, Ali Molloy, Carolin Vegvari

**Affiliations:** 1 Centre for Genomic Pathogen Surveillance, Big Data Institute, University of Oxford, Old Road Campus, Oxford, United Kingdom; 2 Wellcome Genome Campus, Hinxton, United Kingdom; 3 Antimicrobial Resistance Surveillance Reference Laboratory, Research Institute for Tropical Medicine, Muntinlupa, Philippines; 4 Colombian Integrated Program for Antimicrobial Resistance Surveillance–Coipars, CI Tibaitatá, Corporación Colombiana de Investigación Agropecuaria, Tibaitatá–Mosquera, Cundinamarca, Colombia; 5 Global Health Research Unit for the Genomic Surveillance of Antimicrobial Resistance, Department of Pharmaceutical Microbiology, Faculty of Pharmacy, University of Ibadan, Oyo State, Nigeria; 6 Central Research Laboratory, Kempegowda Institute of Medical Sciences, Bengaluru, India

**Keywords:** *Klebsiella*, whole-genome sequencing, WGS, antimicrobial resistance, AMR

## Abstract

In this Supplement, we detail outputs of the National Institute for Health Research Global Health Research Unit on Genomic Surveillance of Antimicrobial Resistance project, covering practical implementation of whole-genome sequencing across our consortium, which consists of laboratories in Colombia, India, Nigeria, and the Philippines.

The promises of whole-genome sequencing (WGS) for surveillance of antimicrobial resistance (AMR) are broad and point toward a revolution in clinical microbiology (eg, for diagnostics and identification of risk to enhance patient management), enhanced insights into transmission dynamics, and allied increased infection control. Furthermore, the ability to observe the underlying evolutionary and population dynamics of pathogens promises to enhance our ability to develop, implement, and monitor interventional approaches such as new antimicrobials and vaccines, as well as nontherapeutic interventions. The collation of WGS data combined with relevant phenotypic, clinical, and epidemiological data at local, regional, national, and international levels will lead to a landscape of data-informed pathogen monitoring, which will enhance public health decision-making and concomitant impact on control.

The potential for us to observe, learn from, and mitigate the risk from the evolutionary and epidemiological dynamics of pathogens is an intensive area of research, and there are increasing bodies of evidence to indicate feasibility and potential routes toward policy and stakeholder impact. However, while the promises are vast, the practicalities of WGS implementation for AMR surveillance are challenging and broad, spanning local and international infrastructure (both laboratory and computational), workforce training and retention, and supply chain and reagent availability, to name a few areas. In addition to practical challenges, we are tasked with the challenges of evolution; pathogens are dynamic entities whose biology changes in adaptation to pressures that we exert as we attempt to mitigate their impact. Policy changes and human behavior can have a direct impact on the evolution of subsets of a pathogen’s population. This has been seen clearly in the current coronavirus 2019 pandemic, where the evolution and spread of particular lineages around the world have been influenced dramatically by the dual impact of movement restrictions and vaccine introductions, and population acceptance of these, largely in countries and locations that can afford such large-scale interventions. There is a pressing need to drive down the time to implementation of methods that will enhance infection control as well as our ability to collate and use data generated to maximum benefit for impact and decision-making at the local, national, and international levels to control AMR.

The National Institute for Health Research Global Health Research Unit (GHRU) on Genomic Surveillance of AMR was initiated to focus on the implementation and integration of WGS within existing or nascent national AMR surveillance systems and to define standard methodologies, technical packages, and workstreams that would enhance the ability to both deliver WGS as a practical local tool and to bring together data and interpretation of pathogen dynamics for local, national, and international utility [1, 2]. In this Supplement, we detail the project’s implementation and outputs, covering practical implementation of WGS across our consortium, which consists of laboratories in Colombia, India, Nigeria, and the Philippines. Each location is at a different stage of national AMR surveillance, and they have different institutional fingerprints. However, our approach has been to collectively identify horizontal technical and data requirements and to develop approaches that can be applied vertically within different backgrounds. Learning and sharing across all contexts has been crucial to the delivery of insight and increased ownership and development of outputs.

Practical implementation of WGS within a microbiology laboratory requires careful consideration and development of protocols, design of laboratory space to optimize practical workflow, and management of both samples and laboratory information in ways that can be maintained over time. Kekre et al detail an iterative approach to implementation of WGS across sites through laboratory assessment, assembly, optimization, and reassessment in order to deliver surveillance capacity [3]. Furthermore, standardization in phenotypic testing allied to standardized genotypic testing is crucial to the uptake and utility of WGS to complement existing and current working laboratory practices.

Concomitant to laboratory capacity, major challenges with WGS implementation are focused on bioinformatics and the ability to rapidly move from raw data through to interpretable information that could be used to understand local dynamics. The development of standardized data pipelines and methods to move directly from sequencer to interpretable outputs—for example, which resistance determinants are seen within which genome and how does this relate to previously seen samples—is the subject of Afolayan et al’s article in which they describe the bottlenecks in bioinformatics deployment and routes to overcome issues which have been implemented as standardized workflows across all GHRU laboratories [4]. Both laboratory methodologies and bioinformatics for WGS are reasonably novel methods that, for broader uptake, require the upskilling of new workforces capable of delivering the value and promise. Each unit within the GHRU acts, to a lesser or greater extent, as a regional hub for expertise. Training, both locally and further afield, can benefit greatly from newer teaching methodologies such as Train-the-Trainer, which aims to enhance pedagogical expertise to deliver training aimed at forward-learning. Our approaches taken within the GHRU but aimed broadly at the use of Train-the-Trainer for genomic pathogen surveillance are detailed by Abrudan et al [5].

An often overlooked aspect within “capacity-building projects” is holistic institutional capacity outside of a specific technology or method. International efforts to standardize aspects of grant management and diligence covered by the Good Financial Grants Practice initiative are detailed by Harste et al, along with standards implementation and accreditation at GHRU sites focused on increasing institutional capacity to accept and manage funds for genomic surveillance, which often requires large transactions and rapid data turnaround [6].

Each partner within the GHRU has priorities with respect to pathogens and questions posed and potentially addressed through the use of WGS, largely driven by local stakeholders and requirements, and local and national pathogen dynamics. In order to provide population-level understanding of pathogens to drive and deliver value for local determination of, for example, hospital-level dynamics, our focus within the GHRU has been on large-scale pathogen surveys aimed at understanding the dynamics of the World Health Organization (WHO) AMR priority pathogens for the generation of new antibiotics, allied to capacity for local prospective sequencing to enhance targeted investigation. The model of longitudinal population snapshots providing insight into dynamics of the clonal structure of pathogens allied to the contextualization of triggered investigations based on phenotypic frequency is investigated and described more fully here related to *Klebsiella pneumoniae*, a pathogen of major importance globally and in each of the GHRU countries. There is significant interest in the development of vaccines for *K. pneumoniae*, and insights into circulating strains and a description of both the clonal population with respect to resistance as well as the distribution of potential vaccine targets (eg, the K/O antigens for *K. pneumoniae*) is crucial to inform local policy and the development of interventions. Prior to these studies, very little was known about the genomic population structure of *K. pneumoniae* in the GHRU countries, and we describe and detail population dynamics at a national and very local scale, as well as the impact of using this information. Saavedra et al describe the complexities of carbapenemase-resistant *Klebsiella* in Colombia and justification for ongoing requirements for surveillance to manage infection control [7]. Similarly, Nagaraj et al apply WGS to detail the emergence of clonal lineages and the impact on requirements for enhanced public health management [8]. Afolayan et al describe *Klebsiella* from Nigeria and identification of facility-level outbreaks of particular clones, while demonstrating the power of WGS to untangle the international dynamics of clones circulating in Nigeria [9]. The power of WGS to aid identification of previously unseen outbreaks is further detailed in the Philippines by Carlos et al, along with the ongoing enhancement of linking phenotypic data for scanning and alerting enhanced by WGS [10]. All of our country-level descriptions focus on the use of WGS for enhanced infection control, insight into local events that can be actioned (eg, outbreak identification and prevention), and the local contextualization of data on national and international circulating lineages. A requirement for bringing data together across nations and delivering value at a very local level while also enabling global-level understanding of pathogen dynamics demands a new breed of data platforms that deliver collation of all available data along with best practice analytics and risk identification (eg, the right AMR determinants and analytic methods). Argimón et al describe the enhancement of such a platform, Pathogenwatch, for the global surveillance of *K. pneumoniae* delivered through a community approach and aimed at democratizing data and insight [11]. Key to this approach is the delivery of value at a local level to enhance understanding at the source of data-generation.

In undertaking this work, we have expanded the geographies of understanding of *Klebsiella* epidemiology, including for the first time data from isolates from multiple institutions from which genomic information was not previously available, recruiting scientists and clinicians working within them to a global endeavor. We have therefore sharpened the understanding of *Klebsiella* pandemic lineages and their distribution very prominently and identified many new ones, illustrating the global value of local genomic surveillance efforts. All of our data are publicly available and therefore usable by others who would like to further contextualize *Klebsiella* genomic epidemiology in their own settings.

While there are considerable additional difficulties in widespread implementation of WGS as a front-line decision-support and surveillance tool, we have detailed technical and practical approaches within the GHRU, exemplified the approach to interpret and understand the dynamics of a major pathogen within our countries, and reported on an approach for ongoing international surveillance through shared and open informatics. Prior to these publications, this work has helped inform (and challenges are described within) the recent WHO publication on the use of WGS for surveillance of AMR within the Global Antimicrobial Resistance Surveillance System [12]. Furthermore, we present our findings along with our lessons as a model for developing-while-doing and to ensure that our experiences as well as our data can be leveraged to promote genomic surveillance in other settings.

Clearly, there are many challenges ahead to achieving the promise of WGS for truly global, local, and international surveillance of AMR. However, barriers are being broken down, and models that practically and tangibly address bottlenecks in local implementation, allied with data collation, linkage, and interpretation methods, both bottom-up and top-down, are likely to drive down implementation time and speed up the delivery of data for action and to enhance, monitor, and adapt to real-time interventional challenges.

## Notes

***Acknowledgments.*** Members of the NIHR Global Health Research Unit on Genomic Surveillance of Antimicrobial Resistance: Khalil Abudahab, Monica Abrudan, Silvia Argimón, Harry Harste, Mihir Kekre, Dawn Muddyman, Ben Taylor, Anthony Underwood, Nicole Wheeler, and Sophia David of the Centre for Genomic Pathogen Surveillance, Big Data Institute, University of Oxford, Old Road Campus, Oxford, United Kingdom and Wellcome Genome Campus, Hinxton, UK. Johan Fabian Bernal, Alejandra Arevalo, Maria Fernanda Valencia, and Erik C. D. Osma Castro of the Colombian Integrated Program for Antimicrobial Resistance Surveillance—Coipars, CI Tibaitatá, Corporación Colombiana de Investigación Agropecuaria, Tibaitatá–Mosquera, Cundinamarca, Colombia. Geetha Nagaraj, Varun Shamanna, Vandana Govindan, Akshata Prabhu, D. Sravani, M. R. Shincy, Steffimole Rose, and Ravishankar K.N. of the Central Research Laboratory, Kempegowda Institute of Medical Sciences, Bengaluru, India. Anderson O. Oaikhena, Ayorinde O. Afolayan, Jolaade J Ajiboye, and Erkison Ewomazino Odih of the Department of Pharmaceutical Microbiology, Faculty of Pharmacy, University of Ibadan, Oyo State, Nigeria. Marietta L. Lagrada, Polle Krystle V. Macaranas, Agnettah M. Olorosa, June M. Gayeta, and Elmer M. Herrera of the Antimicrobial Resistance Surveillance Reference Laboratory, Research Institute for Tropical Medicine, Muntinlupa, the Philippines. Ali Molloy, alimolloy.com; John Stelling, Brigham and Women’s Hospital; and Carolin Vegvari, Imperial College London.

***Disclaimer.*** This research was commissioned by the National Institute for Health Research (NIHR) using Official Development Assistance (ODA) funding. The views expressed in this publication are those of the authors and not necessarily those of the NHS, NIHR, or Department of Health.

***Financial support.*** This work was supported by ODA funding from the NIHR (grant16_136_111). I. N. O. is supported by an African Research Leader Award from the UK Medical Research Council (MRC) and the UK Department for International Development (DFID) under the MRC/DFID Concordat agreement that is also part of the European & Developing Countries Clinical Trials Partnership program supported by the European Union (I. N. O., University of Ibadan; pathogenic lineages of enteric bacteria in Nigeria MR/L00464X/1). K. L. R. reports travel support from NIHR, UK.

***Supplement sponsorship.*** The supplement is sponsored by the UK National Institute for Health Research Global Health Research Unit on Genomic Surveillance of AMR.

***Potential conflicts of interest.*** I. N. O. is a member of the Bill & Melinda Gates Foundation Surveillance Advisors Group (from 2019) and has received grants and nonfinancial support from the International Vaccine Institute (Severe Typhoid Fever Surveillance in Africa program Award to the University of Ibadan). All remaining authors: No reported conflicts of interest. All authors have submitted the ICMJE Form for Disclosure of Potential Conflicts of Interest. Conflicts that the editors consider relevant to the content of the manuscript have been disclosed.
